# The Effect of Heated, Humidified High‐Flow Air in COPD Patients With Chronic Bronchitis

**DOI:** 10.1155/pm/8350741

**Published:** 2025-12-28

**Authors:** Spyridon Fortis, Eric A. Hoffman, Alejandro P. Comellas

**Affiliations:** ^1^ Division of Pulmonary, Critical Care and Occupational Medicine, University of Iowa Hospital and Clinics, Iowa City, Iowa, USA, uihealthcare.org; ^2^ Center for Access & Delivery Research & Evaluation (CADRE), Iowa City VA Health Care System, Iowa City, Iowa, USA; ^3^ Roy J. Carver Department of Biomedical Engineering, University of Iowa, Iowa City, Iowa, USA, uiowa.edu; ^4^ Department of Radiology, University of Iowa, Iowa City, Iowa, USA, uiowa.edu

**Keywords:** chronic obstructive, death, disease progression, emphysema, pulmonary disease, smoking

## Abstract

**Introduction:**

Heated, humidified high‐flow air (HHHFA) has been shown to reduce exacerbations in patients with COPD or bronchiectasis with significant sputum production. This pilot study evaluated the short‐term effects of nocturnal HHHFA in COPD patients with chronic bronchitis.

**Methods:**

This was a prospective, single‐center, open‐label, randomized, placebo‐controlled trial. Participants with COPD, chronic bronchitis, and ≥ 2 exacerbations in the prior year were randomized to either nocturnal HHHFA or usual care. Assessments included sleep quality, dyspnea, quality of life, cough, lung function, imaging, and exercise capacity at baseline and 6 weeks.

**Results:**

Of 11 eligible participants, seven completed the study (four intervention, three control). Baseline characteristics were generally similar, though the intervention group had a higher BMI and a lower emphysema percentage. No statistically significant differences were observed between groups in primary or secondary outcomes.

**Conclusions:**

Nocturnal HHHFA over 6 weeks did not improve clinical or imaging outcomes in this small cohort of COPD patients with chronic bronchitis. The study was likely underpowered due to recruitment challenges. Larger trials are needed to assess the potential of HHHFA in this population.

**Trial Registration:**

ClinicalTrials.gov identifier: NCT03959982

## 1. Introduction

Chronic obstructive pulmonary disease (COPD) is marked by exacerbations that increase in frequency with disease severity and often lead to hospitalizations—the primary driver of COPD‐related healthcare costs and a major burden for patients and families [1]. Chronic bronchitis, defined by persistent cough and sputum production for at least 3 months in 2 consecutive years, doubles the risk of exacerbations and hospitalizations [2]. It is also linked to worse dyspnea, reduced quality of life and sleep, and impaired exercise capacity due to air trapping and hyperinflation [2]. Despite its impact, treatment options beyond standard inhaled therapies remain limited.

Heated, humidified high‐flow air (HHHFA) has been shown to reduce exacerbations in patients with COPD or bronchiectasis with significant sputum production [3]. This pilot study evaluates the effect of nocturnal HHHFA in COPD patients with chronic bronchitis, focusing on short‐term outcomes including respiratory symptoms, quality of life, sleep, lung function, imaging features, and exercise capacity.

## 2. Methods

This was a prospective, single‐center, open‐label, randomized, placebo‐controlled trial. The study protocol was approved by the institutional review board (IRB #201905817;) Participants were eligible if they had COPD, defined as a post‐bronchodilator FEV₁/FVC ratio < 0.7 and a smoking history of ≥ 10 pack‐years, with a post‐bronchodilator FEV₁% predicted < 70%, chronic bronchitis, and ≥ 2 exacerbations in the previous year. Exclusion criteria included confirmed or suspected sleep apnea requiring positive airway pressure therapy, continuous oxygen supplementation, recent (< 2 weeks) respiratory infection, COPD exacerbation, acute bronchitis requiring antibiotics and systemic corticosteroids, upper airway or chest surgery within the past 6 months, pregnancy, and any condition that prevented the participant from wearing the HHHFA nasal cannula or completing study procedures. Participant recruitment began in February 2021 and was discontinued in January 2023 due to under recruitment. Eligible participants provided written informed consent and were then randomized to either the HHHFA intervention arm or the usual care arm using sealed envelopes. At baseline, participants completed the following assessments: Pittsburgh Sleep Quality Index (PSQI) [4], Medical Research Council (MRC) dyspnea scale [5], St. George′s Respiratory Questionnaire (SGRQ) [6–8], COPD assessment test (CAT) [9], and cough and sputum assessment questionnaire (CASA‐Q) [10]. They also underwent post‐bronchodilator spirometry, a 6‐minute walk test, and inspiratory and expiratory chest CT scans. These procedures were repeated at the 6‐week follow‐up visit.

### 2.1. Intervention

Participants assigned to the HHHFA arm trialed the device during the initial visit. Temperature and flow settings were adjusted for comfort within the allowable range of 32°C–37°C and 20–35 L/min, as previously described. Participants were instructed to use the device for ≥ 4 h during sleep. Compliance was verified using the device′s usage log.

### 2.2. Definitions

Functional small airway disease was quantified using the disease probability method (DPM^FSAD^) [11]. Changes in PSQI, mMRC, SGRQ, CAT, CASA‐Q scores, 6‐minute walk distance, FEV₁, RV, TLV, RV/TLC, % emphysema, % gas trapping, % DPM^FSAD^, and Pi10 (mm) were calculated as the difference between baseline and the 6‐week follow‐up visit. Positive values indicated higher measurements at follow‐up.

### 2.3. Statistical Analysis

Participants were grouped based on intervention status. Baseline characteristics were compared using the Wilcoxon rank‐sum test for continuous variables and Fisher′s exact test for categorical variables. Due to the small sample size and imbalance between groups despite randomization, we used linear regression models to assess associations between the intervention and primary/secondary outcomes, adjusting for baseline values (e.g., baseline PSQI). For secondary outcomes, we applied the Benjamini–Hochberg correction with a false discovery rate threshold of < 0.05.

## 3. Results

Of the 11 patients who met the eligibility criteria, two withdrew from the study. One withdrew prior to randomization due to a new diagnosis of lung cancer, and one participant randomized to the intervention arm dropped out before completing the study procedures. Among the remaining seven participants, four were assigned to the intervention group and three to the control group. Baseline characteristics were generally comparable (Table 1), except for two notable differences: participants in the intervention group had a significantly higher body mass index (BMI: 32.94 [interquartile interval (IQI): 29.61, 36.56]) compared with those in the control group (24.18 [IQI: 23.98, 25.57]; *p* = 0.034), and a lower percentage of emphysema (2.80% [IQI: 2.13, 3.37] vs. 14.57% [IQI: 10.29, 14.86]; *p* = <0.034). The primary outcome was not achieved, and none of the secondary outcomes showed statistically significant differences between groups (Table 2).

**Table 1:** Baseline characteristics.

	**Control**	**HHHFA**	**p** **value**
*n*	3	4	
Age	61.00 [57.50, 70.00]	58.00 [56.50, 63.00]	0.72
Female	3 (100.0%)	2 (50.0%)	0.55
Race	3 (100.0%)	4 (100.0%)	NA
Body mass index, Kg/m^2^	24.18 [23.98, 25.57]	32.94 [29.61, 36.56]	0.034
Pack years	7.50 [4.75, 10.12]	12.25 [3.39, 27.88]	0.72
Current smoking	1 (33.3%)	3 (75.0)	0.74
Asthma	2 (66.7)	3 (75.0)	1
PSQI	11.00 [8.50, 11.00]	17.00 [15.25, 17.25]	0.15
MMRC	1.00 [0.50, 2.00]	2.50 [2.00, 3.25]	0.21
SGRQ	59.99 [48.17, 68.81]	70.81 [66.41, 72.67]	0.72
CAT	35.00 [32.00, 36.50]	32.00 [31.00, 33.00]	0.48
CASA‐Q cough	83.00 [66.50, 87.50]	66.50 [56.00, 81.25]	0.86
CASA‐Q impact	63.00 [47.00, 73.00]	62.50 [49.25, 77.25]	0.72
CASA‐Q sputum	75.00 [62.50, 83.50]	67.00 [67.00, 73.25]	0.86
6‐min walk distance, m	373.4 [365.0, 376.4]	245.9 [142.8, 340.2]	0.034
FEV_1_, L	1.14 [1.03, 1.77]	1.47 [1.38, 1.58]	0.72
FEV_1_% predicted	55.49 [46.02, 63.56]	54.64 [45.27, 61.56]	1
FVC, L	3.04 [2.87, 3.52]	3.12 [2.62, 3.79]	1
FVC % predicted	94.41 [94.05, 96.92]	91.18 [68.32, 113.59]	1
FEV_1_/FVC	42.00 [36.00, 51.00]	41.50 [39.50, 49.75]	0.72
RV, L	3.96 [3.02, 4.11]	3.41 [3.16, 3.50]	0.48
TLC, L	5.68 [5.25, 6.08]	5.38 [4.62, 6.32]	0.72
RV/TLC	65.55 [51.10, 73.80]	54.90 [52.49, 63.15]	1
% Emphysema	14.57 [10.29, 14.86]	2.80 [2.13, 3.37]	0.034
% Gas Trapping	56.81 [33.76, 59.22]	21.28 [17.45, 24.47]	0.48
% DPM^FSAD^	23.99 [14.92, 29.01]	6.33 [4.08, 7.59]	0.1657
Pi10, mm	3.96 [3.95, 3.98]	3.95 [3.94, 3.95]	0.48

Abbreviations: CASA‐Q cough = cough domain of the cough and sputum assessment questionnaire; CASA‐Q impact = impact domain of the cough and sputum assessment questionnaire; CASA‐Q sputum = sputum domain of the cough and sputum assessment questionnaire; CAT = COPD assessment test; % DPMFSAD = percentage of disease probability map functional small airways disease; FEV₁ = forced expiratory volume in 1 s; FEV₁/FVC = ratio of FEV₁ to forced vital capacity; FVC = forced vital capacity; mMRC = modified Medical Research Council dyspnea scale; PSQI = Pittsburgh Sleep Quality Index; RV = residual volume; RV/TLC = ratio of residual volume to total lung capacity; SGRQ = St. George′s Respiratory Questionnaire; TLC = total lung capacity.

**Table 2:** Association of heated, humidified high‐flow air with longitudinal outcome.

**Change between baseline and 6-week follow-up visit**	**Coef (95% CI)**	**p** **value**	**Adjusted** **p** **value**
PSQI∗	2.19 (−7.945, 12.3)	0.58	**NA**
MMRC	1.04 (0.03, 2.06)	0.05	0.32
CAT	5.15 (−3.01, 13.32)	0.16	0.54
SGRQ	14.83 (0.41, 29.25)	0.05	0.65
Casa‐Q cough domain	5.28 (−30.83, 41.39)	0.71	1.00
Casa‐Q impact	1.84 (−36.14, 39.82)	0.90	0.97
Casa‐Q sputum	−8.22 (−32.71, 16.28)	0.40	0.81
6‐min walk distance, m	−41.72 (−163.57, 80.13)	0.40	0.92
FEV_1_, mL	−54.49 (−681.4, 572.4)	0.82	0.96
RV, L	60.4 (−577.2, 698.1)	0.81	1.00
TLC, L	70.6 (−494.3, 635.5)	0.75	1.00
% Emphysema	−3.66 (−10.32, 2.99)	0.20	0.56
% Gas trapping	1.44 (−41.39, 44.27)	0.93	0.93
DPM^FSAD^	−3.75 (−21.11, 13.60)	0.58	1.00
Pi10, m	0.07 (−0.03, 0.17)	0.11	0.52

∗Primary outcome.

Abbreviations: CASA‐Q cough = cough domain of the cough and sputum assessment questionnaire; CASA‐Q impact = impact domain of the cough and sputum assessment questionnaire; CASA‐Q sputum = sputum domain of the cough and sputum assessment questionnaire; CAT = COPD assessment test; DPMFSAD = percentage of disease probability map functional small airways disease ;FEV₁ = forced expiratory volume in 1 s; mMRC = modified Medical Research Council dyspnea scale; PSQI = Pittsburgh Sleep Quality Index; RV = residual volume; RV/TLC = ratio of residual volume to total lung capacity; SGRQ = St. George′s Respiratory Questionnaire; TLC = total lung capacity.

## 4. Discussion

In this single‐center study, we found that nocturnal use of HHHFA over a 6‐week period in patients with COPD and chronic bronchitis was not associated with improvements in sleep quality, dyspnea, cough, health‐related quality of life, exercise capacity, or CT‐based features of obstructive lung disease. These findings are likely attributable to the study being underpowered.

HHHFA is a well‐established treatment for acute hypoxemic respiratory failure [12]. More recently, it has demonstrated benefits in acute hypercapnic respiratory failure due to COPD exacerbations [13]. In patients with chronic hypercapnic respiratory failure secondary to COPD, HHHFA has been shown to reduce exacerbations and improve health‐related quality of life [14–16]. Additionally, in patients with COPD or bronchiectasis who experience a significant burden of chronic bronchitis (defined as ≥ 5 mL of daily sputum production), even short daily use of HHHFA (2 h) has been shown to reduce exacerbations and improve lung function [3].

In our study, however, no significant associations were observed—likely due to limited statistical power. Recruitment was particularly challenging, as the study was conducted during the COVID‐19 pandemic, which not only disrupted clinical research operations but also disproportionately affected patients with COPD [17–20]. These factors likely contributed to the difficulty in identifying and enrolling eligible participants and underscored the need for broader inclusion criteria in future trials. In addition, the strict inclusion and exclusion criteria—such as excluding patients on continuous oxygen therapy or those with sleep apnea—made enrollment particularly challenging. These limitations were compounded by the inherent difficulties of recruiting patients for home‐based respiratory device studies, where adherence to study protocols is often problematic. A recent study involving patients with hypercapnic respiratory failure due to COPD, recruited after hospital discharge, encountered similar recruitment barriers. Notably, patients assigned to HHHFA experienced reductions in nocturnal transcutaneous CO_2_ (PtcCO_2_) levels and improvements in health‐related quality of life [21]. A feasibility study similar to ours, which recruited patients upon discharge following a COPD exacerbation, showed trends toward improvement in clinical outcomes [22].

In summary, nocturnal HHHFA use over a 6‐week period in patients with COPD and chronic bronchitis did not improve sleep quality or other clinical outcomes in our study, which was likely underpowered. Future studies should target this population with larger sample sizes and broader inclusion criteria to better evaluate the potential of HHHFA in reducing the burden of COPD exacerbations.

## Disclosure

VIDA, Astra Zeneca, GSK, and Fisher and Paykel Healthcare had no role in study design, data collection and analysis, decision to publish, or preparation of the manuscript. They had no impact on the outcome of the clinical reports collection. Spyridon Fortis had full access to all data in the study and takes responsibility for the integrity of the data and the accuracy of the data analysis.

## Conflicts of Interest

S.F. is supported by the Office of Rural Health and has served as a consultant for the Society of Hospital Medicine (SHM), and has stock options with ROMTech. E.A.H. is a founder and shareholder of VIDA Diagnostics. A.P.C. has served as a consultant for VIDA, Astra Zeneca, and GSK.

## Author Contributions

Study concept: S.F., E.A.H., and A.P.C. Study design: S.F., E.A.H., and A.P.C. Acquisition, analysis, or interpretation of data: all authors. Drafting of the manuscript: S.F. Critical revision of the manuscript for important intellectual content: all authors.

## Funding

This study was supported by the ATS Foundation/Fisher and Paykel Healthcare Ltd. Research Award (ATS grant # RP‐2018‐05).

## General Statement

*Disclaimer*: The views expressed in this article are those of the authors and do not necessarily reflect the position or policy of the Department of Veterans Affairs or the United States Government and of the National Institutes of Health′s National Center for Advancing Translational Sciences.

## Data Availability

The data that support the findings of this study are available on request from the corresponding author. The data are not publicly available due to privacy or ethical restrictions.

## References

[1] Celli B. R. and Wedzicha J. A. , Update on Clinical Aspects of Chronic Obstructive Pulmonary Disease, New England Journal of Medicine. (2019) 381, no. 13, 1257–1266, 10.1056/NEJMra1900500, 2-s2.0-85072654950, 31553837. 31553837

[2] Kim V. and Criner G. J. , Chronic Bronchitis and Chronic Obstructive Pulmonary Disease, American Journal of Respiratory and Critical Care Medicine. (2013) 187, no. 3, 228–237, 10.1164/rccm.201210-1843CI, 2-s2.0-84873353231, 23204254. 23204254 PMC4951627

[3] Rea H. , McAuley S. , Jayaram L. , Garrett J. , Hockey H. , Storey L. , O′Donnell G. , Haru L. , Payton M. , and O′Donnell K. , The Clinical Utility of Long-Term Humidification Therapy in Chronic Airway Disease, Respiratory Medicine. (2010) 104, no. 4, 525–533, 10.1016/j.rmed.2009.12.016, 2-s2.0-77349126034, 20144858. 20144858

[4] Buysse D. J. , Reynolds C. F.3rd, Monk T. H. , Berman S. R. , and Kupfer D. J. , The Pittsburgh Sleep Quality Index: A New Instrument for Psychiatric Practice and Research, Psychiatry Research. (1989) 28, no. 2, 193–213, 10.1016/0165-1781(89)90047-4, 2-s2.0-0024389366, 2748771. 2748771

[5] Bestall J. C. , Paul E. A. , Garrod R. , Garnham R. , Jones P. W. , and Wedzicha J. A. , Usefulness of the Medical Research Council (MRC) Dyspnoea Scale as a Measure of Disability in Patients With Chronic Obstructive Pulmonary Disease, Thorax. (1999) 54, no. 7, 581–586, 10.1136/thx.54.7.581, 2-s2.0-0008485492, 10377201. 10377201 PMC1745516

[6] Meguro M. , Barley E. A. , Spencer S. , and Jones P. W. , Development and Validation of an Improved, COPD-Specific Version of the St. George Respiratory Questionnaire, Chest. (2007) 132, no. 2, 456–463, 10.1378/chest.06-0702, 2-s2.0-34548031399, 17646240. 17646240

[7] Jones P. W. , Quirk F. H. , and Baveystock C. M. , The St George′s Respiratory Questionnaire, Respiratory Medicine. (1991) 85, 25–31, 10.1016/S0954-6111(06)80166-6, 2-s2.0-0025886867. 1759018

[8] Jones P. W. , Quirk F. H. , Baveystock C. M. , and Littlejohns P. , A Self-Complete Measure of Health Status for Chronic Airflow Limitation. The St. George′s Respiratory Questionnaire, American Review of Respiratory Disease. (1992) 145, no. 6, 1321–1327, 10.1164/ajrccm/145.6.1321, 2-s2.0-0026871146, 1595997. 1595997

[9] Jones P. W. , Harding G. , Berry P. , Wiklund I. , Chen W. H. , and Kline L. N. , Development and First Validation of the COPD Assessment Test, European Respiratory Journal. (2009) 34, no. 3, 648–654, 10.1183/09031936.00102509, 2-s2.0-70349097551. 19720809

[10] Crawford B. , Monz B. , Hohlfeld J. , Roche N. , Rubin B. , Magnussen H. , Nivens C. , Ghafouri M. , McDonald J. , and Tetzlaff K. , Development and Validation of a Cough and Sputum Assessment Questionnaire, Respiratory Medicine. (2008) 102, no. 11, 1545–1555, 10.1016/j.rmed.2008.06.009, 2-s2.0-55549091050. 18662868

[11] Fortis S. , Comellas A. P. , and Hoffman E. A. , Advances in the Characterisation of COPD Using Quantitative Imaging, COPD in the 21st Century, 2024, European Respiratory Society, 10.1183/2312508X.10006523.

[12] Frat J. P. , Thille A. W. , Mercat A. , Girault C. , Ragot S. , Perbet S. , Prat G. , Boulain T. , Morawiec E. , Cottereau A. , Devaquet J. , Nseir S. , Razazi K. , Mira J. P. , Argaud L. , Chakarian J. C. , Ricard J. D. , Wittebole X. , Chevalier S. , Herbland A. , Fartoukh M. , Constantin J. M. , Tonnelier J. M. , Pierrot M. , Mathonnet A. , Béduneau G. , Delétage-Métreau C. , Richard J. C. M. , Brochard L. , and Robert R. , High-Flow Oxygen Through Nasal Cannula in Acute Hypoxemic Respiratory Failure, New England Journal of Medicine. (2015) 372, no. 23, 2185–2196, 10.1056/NEJMoa1503326, 2-s2.0-84930535780. 25981908

[13] RENOVATE Investigators and the BRICNet , High-Flow Nasal Oxygen vs. Noninvasive Ventilation in Patients With Acute Respiratory Failure: The RENOVATE Randomized Clinical Trial, JAMA. (2025) 333, no. 10, 875–890, 10.1001/jama.2024.26244, 39657981. 39657981 PMC11897836

[14] Nagata K. , Horie T. , Chohnabayashi N. , Jinta T. , Tsugitomi R. , Shiraki A. , Tokioka F. , Kadowaki T. , Watanabe A. , Fukui M. , Kitajima T. , Sato S. , Tsuda T. , Kishimoto N. , Kita H. , Mori Y. , Nakayama M. , Takahashi K. , Tsuboi T. , Yoshida M. , Hataji O. , Fuke S. , Kagajo M. , Nishine H. , Kobayashi H. , Nakamura H. , Okuda M. , Tachibana S. , Takata S. , Osoreda H. , Minami K. , Nishimura T. , Ishida T. , Terada J. , Takeuchi N. , Kohashi Y. , Inoue H. , Nakagawa Y. , Kikuchi T. , and Tomii K. , Home High-Flow Nasal Cannula Oxygen Therapy for Stable Hypercapnic COPD: A Randomized Clinical Trial, American Journal of Respiratory and Critical Care Medicine. (2022) 206, no. 11, 1326–1335, 10.1164/rccm.202201-0199OC, 35771533. 35771533 PMC9746854

[15] Theunisse C. , de Graaf N. T. C. , Braam A. W. E. , Vonk G. C. , Baart S. J. , Ponssen H. H. , and Cheung D. , The Effects of Home High-Flow Nasal Cannula Oxygen Therapy on Clinical Outcomes in Patients With Severe COPD and Frequent Exacerbations, Journal of Clinical Medicine. (2025) 14, no. 3, 10.3390/jcm14030868, 39941539. PMC11818408 39941539

[16] Nagata K. , Kikuchi T. , Horie T. , Shiraki A. , Kitajima T. , Kadowaki T. , Tokioka F. , Chohnabayashi N. , Watanabe A. , Sato S. , and Tomii K. , Domiciliary High-Flow Nasal Cannula Oxygen Therapy for Patients With Stable Hypercapnic Chronic Obstructive Pulmonary Disease. A Multicenter Randomized Crossover Trial, Annals of the American Thoracic Society. (2018) 15, no. 4, 432–439, 10.1513/AnnalsATS.201706-425OC, 2-s2.0-85045403608, 29283682. 29283682

[17] Muneeb Hassan M. , Ameeq M. , Jamal F. , Tahir M. H. , and Mendy J. T. , Prevalence of Covid-19 Among Patients With Chronic Obstructive Pulmonary Disease and Tuberculosis, Annals of Medicine. (2023) 55, no. 1, 285–291, 10.1080/07853890.2022.2160491, 36594409. 36594409 PMC9815254

[18] Hassan M. M. , Tahir M. H. , Ameeq M. , Jamal F. , Mendy J. T. , and Chesneau C. , Risk Factors Identification of COVID-19 Patients With Chronic Obstructive Pulmonary Disease: A Retrospective Study in Punjab-Pakistan, Immunity, Inflammation and Disease. (2023) 11, no. 8, e981, 10.1002/iid3.981, 37647450. 37647450 PMC10461420

[19] Hassan M. M. , Sikandar S. M. , Jamal F. , Ameeq M. , and Kargbo A. , The Complex Relationship Between Chronic Obstructive Pulmonary Disease With Cardiovascular Disease and Their Interactions With COVID-19 Vaccination: A Retrospective Study, Immunity, Inflammation and Disease. (2024) 12, no. 11, e70068, 10.1002/iid3.70068, 39555721. 39555721 PMC11571095

[20] Hassan M. M. , Sikandar S. M. , Jamal F. , Ameeq M. , and Kargbo A. , Chronic Obstructive Pulmonary Disease Patients With Community-Acquired Pneumonia on Inhaled Corticosteroid Therapy: A Comprehensive Analysis of Risk Factors, Disease Burden, and Prevention Strategies, Health Science Reports. (2025) 8, no. 1, e70395, 10.1002/hsr2.70395, 39872908. 39872908 PMC11770223

[21] Nilius G. , Schroeder M. , Domanski U. , Khalaf M. , and Tatkov S. , Nocturnal Respiratory Support With Nasal High Flow in Hypercapnic COPD: A Randomised, Crossover Trial, ERJ Open Research. (2025) 11, no. 3, 01063–02024, 10.1183/23120541.01063-2024, 40589905. 40589905 PMC12208599

[22] Pandya A. A. , Criner L. H. , Thomas J. , Jacobs M. , and Criner G. J. , Tolerability and Safety of High-Flow Nasal Therapy in Patients Hospitalized With an Exacerbation of COPD, Chronic Obstructive Pulmonary Diseases: Journal of the COPD Foundation. (2020) 7, no. 4, 362–369, 10.15326/jcopdf.7.4.2020.0137, 32926607. 32926607 PMC7883908

